# The herbicide glyphosate inhibits hippocampal long-term potentiation and learning through activation of pro-inflammatory signaling

**DOI:** 10.1038/s41598-023-44121-7

**Published:** 2023-10-21

**Authors:** Yukitoshi Izumi, Kazuko A. O’Dell, Charles F. Zorumski

**Affiliations:** 1 Department of Psychiatry, Washington University School of Medicine, St. Louis, MO USA; 2 The Taylor Family Institute for Innovative Psychiatric Research, Washington University School of Medicine, St. Louis, MO USA

**Keywords:** Cognitive neuroscience, Learning and memory

## Abstract

Glyphosate, a herbicide marketed as Roundup, is widely used but there are concerns this exposure could impair cognitive function. In the CA1 region of rat hippocampal slices, we investigated whether glyphosate alters synaptic transmission and long-term potentiation (LTP), a cellular model of learning and memory. Our hypothesis is that glyphosate alters neuronal function and impairs LTP induction via activation of pro-inflammatory processes. Roundup depressed excitatory synaptic potentials(EPSPs) in a dose-dependent manner with complete suppression at 2000 mg/L. At concentrations ≤ 20 mg/L Roundup did not affect basal transmission, but 4 mg/L Roundup administered for 30 min inhibited LTP induction. Acute administration of 10–100 μM glyphosate also inhibited LTP induction. Minocycline, an inhibitor of microglial activation, and TAK-242, an inhibitor of toll-like receptor 4 (TLR4), both overcame the inhibitory effects of 100 µM glyphosate. Similarly, lipopolysaccharide from Rhodobacter sphaeroides (LPS-RS), a different TLR4 antagonist, overcame the inhibitory effects. In addition, ISRIB (integrated stress response inhibitor) and quercetin, an inhibitor of endoplasmic reticulum stress, overcame the inhibitory effects. We also observed that in vivo glyphosate injection (16.9 mg/kg i.p.) impaired one-trial inhibitory avoidance learning. This learning deficit was overcome by TAK-242. These observations indicate that glyphosate can impair cognitive function through pro-inflammatory signaling in microglia.

## Introduction

Glyphosate, which was first developed in 1950, was originally used as a descaling agent to clean pipes in the 1960’s. Because glyphosate inhibits the plant enzyme 5-enolpyruvylshikimate-3-phosphate synthase in the aromatic amino acid biosynthetic pathway, it was patented as a herbicide in 1970 and brought to market under the trade name Roundup. The market for glyphosate expanded exponentially as genetically modified plants were developed in the 1990’s. Accordingly, human exposure to glyphosate has become routine across populations. Glyphosate is commonly detected in water samples of streams in the US (median 0.05 µg/L, maximal 8.1 µg/L)^1^. In France, it was detected in over 99% of human urine samples^2^ while in central India, glyphosate was detected in 93% of the urine samples with a mean (SD) concentration of 3.4 (1.2) µg/L^3^. Because only 1% of glyphosate is secreted in urine^4^, this level indicates possibly significant exposure.

Given this vast exposure, a critical question is whether glyphosate is toxic. In addition to its potential carcinogenicity including non-Hodgkin’s lymphoma and hepatic cancer^5^, some studies have linked glyphosate with autism spectrum disorder (ASD)^6^. In mice, maternal glyphosate exposure results in abnormal behaviors and growth retardation in offspring when dams received seven injections of glyphosate (24 or 35 mg/kg) over 2 weeks^7^, or drank water containing about 1 mg/L GBH (glyphosate-based herbicide) for 2 weeks^8^, implying that exposure to glyphosate alters neuronal function directly or indirectly. Additionally, acute exposure of rats to glyphosate decreases monoamine levels in brain^9,10^ supporting a possible link to Parkinson’s disease (PD)^11^. Oral exposure of glyphosate to mice (250 or 500 mg/kg/day^12^ or 1% GBH in drinking water of pregnant rats^13^) is reported to cause depression-like behaviors. Moreover, glyphosate exposure (250 or 500 mg/kg/day of GBH as oral gavages^14^) may diminish memory formation in mice. Although it is plausible that these neuronal sequelae are at least partially induced indirectly by intestinal microbial degradation^15^, it is also possible that the herbicide directly impairs neuronal function because glyphosate passes the blood brain barrier (BBB)^16^ and infiltrates the brain to induce neuroinflammation^17–19^.

Using ex vivo rat hippocampal slices, we investigated whether glyphosate administered directly onto brain tissue alters synaptic transmission and long-term potentiation (LTP), a form of synaptic plasticity thought to contribute to learning and memory. We also examined whether activation of neural pro-inflammatory processes contributes to effects of glyphosate on hippocampal function. To investigate whether glyphosate alters memory formation we tested rats for memory acquisition in a one-trial inhibition avoidance learning task previously linked to hippocampal LTP^20^.

## Methods

### Animals

Sprague–Dawley albino rats were offspring of pregnant female rats obtained from Charles River Laboratories (Indianapolis IN) and were housed in approved facilities at Washington University. Animal use followed National Institute of Health (NIH) guidelines and was approved by the Washington University Institutional Animal Care and Use Committee (IACUC). The reporting in this manuscript follows recommendations in the ARRIVE guidelines.

### Hippocampal slice preparation and physiology

Hippocampal slices were prepared from postnatal day (P) 28–32 male albino rats using previously described methods^21,22^. Dissected hippocampi were pinned on an agar base in ice-cold artificial cerebrospinal fluid (ACSF) containing (in mM): 124 NaCl, 5 KCl, 2 MgSO_4_, 2 CaCl_2_, 1.25 NaH_2_PO_4_, 22 NaHCO_3_, 10 glucose, bubbled with 95% O_2_–5% CO_2_ at 4–6 °C. The dorsal two-thirds of the hippocampus was cut into 500 µm slices using a rotary slicer^23^. Acutely prepared slices were kept in an incubation chamber containing gassed ACSF for at least 1 h at 30 °C before experiments.

For electrophysiological studies, slices were transferred to a submersion-recording chamber at 30 °C with ACSF and perfused continuously at 2 ml/min. Extracellular recordings were obtained from the apical dendritic layer (*stratum radiatum*) of area CA1 for monitoring excitatory postsynaptic potentials (EPSPs) with electrodes filled with 2 M NaCl (5–10 MΩ resistance).

Because LTP is a synaptic phenomenon, we focused on recordings of EPSP slope. EPSPs were evoked using 0.1 ms constant current pulses through a bipolar stimulating electrode in the Schaffer collateral (SC) pathway. Responses were monitored by applying single stimuli every 60 s at half-maximal intensity based on a control input–output (IO) curve (see Supplemental Fig. 1). After obtaining stable baseline recordings for at least 10 min, LTP was induced by a single 100 Hz × 1 s high frequency stimulation (HFS) using the same intensity stimulus. We chose this form of HFS because it induces reliable and stable LTP in our slices with sufficient dynamic range for pharmacological studies. Following HFS, responses were monitored by single stimuli once per minute during the period of post-tetanic potentiation (PTP) and then every five minutes for the remainder of an experiment. For display purposes, graphs show data every 5 min except during initial post-tetanic potentiation.

### In vivo injection of glyphosate and behavioral studies

Rats were tested for memory acquisition in a one-trial inhibitory avoidance learning task^20,24,25^. This task reflects explicit-declarative fear memories and has been associated with hippocampal LTP; the task is relatively simple to administer with high reliability and clear behavioral endpoints^21,22,26,27^. The testing apparatus consists of two chambers, only one of which is lit. Both compartments have a floor of stainless steel rods (4 mm diameter, spaced 10 mm apart) through which an electrical shock can be delivered in the dark chamber (12 × 20 × 16 cm). The adjoining lit compartment (30 × 20 × 16 cm) was illuminated with four 13 W lights. Light intensity in the lit chamber was 1000 lx while that in the dark chamber was < 10 lx. On the first day of testing, rats were brought to the lab for vehicle injection, placed in the lit chamber, and allowed to habituate to the apparatus by freely moving between chambers for 10 min without any foot shocks being administered. On the next day, rats were administered glyphosate (16.9 mg/kg ip) or vehicle (saline) 1 h prior to training. TAK-242 (3 mg/kg i.p.) was injected 24 h and 2 h before glyphosate administration. The dose of glyphosate for in vivo studies was based on the concentration that immediately blocks LTP induction in ex vivo experiments, but is lower than the dose (75 mg/kg) that has been shown to disrupt motor coordination^28^. The dose of glyphosate is also below the reported dose that induces genotoxicity in rats. The dose of TAK-242 is based on a previous report that successfully revealed neuroprotection^29^. At the time of training, animals were initially placed in the lit compartment and allowed to explore the apparatus freely for up to 300 s (5 min). When rats completely entered the dark chamber, they were immediately given a foot shock. After each 300 s session, rats were removed from the apparatus and returned to their home cages. On the next day of testing, rats were placed in the lit chamber without any drug treatment and the latency to enter the dark compartment was recorded over a 300 s trial.

### Chemicals

TAK-242 (CAS 243984-11-4 Cat 6587) was purchased from R&D Systems (Minneapolis MN). Lipopolysaccharide from Rhodobacter sphaeroides (LPS-RS) (Catalog # tlrl-rslps) and MCC950 (CAS 210826-40-7, Catalog # inh-mcc) were purchased from InvivoGen (San Diego CA). Trans-ISRIB (CAS 1597403-47-8, Cat 5284) was from Tocris (Ellisville MO). Other chemicals, including glyphosate (CAS 1071-83-6), minocycline (CAS 13614-98-7, Cat# M2280000) and IL1-Ra (Cat# SRP 3084), quercetin (CAS 849061-97-8, PHR1488) and salts were obtained from Millipore Sigma Chemical Company. Roundup, a herbicide containing glyphosate, was purchased from a local store. Drugs were prepared as stock solutions in either ACSF or DMSO and diluted to final concentration at the time of experiment. The concentrations of TAK-242, LPS-RS and minocycline are based on our previous studies using those inhibitors against lipopolysaccharide (LPS) and acrylamide^21,22^. The concentrations of MCC950 were also based on our previous paper^22^. The dose of TAK-242 in the behavioral study followed a proceeding report by Ono et al.^30^.

### Statistical analysis

Physiological data were collected and analyzed using PClamp software (Molecular Devices, San Jose CA). Data are expressed as mean ± SEM 60 min following HFS, and are normalized with respect to initial baseline recordings (taken as 100%). N of slices is identical to that of animals unless stated otherwise. Statistical comparisons in physiological studies were based on IO curves at baseline and sixty minutes following HFS to determine the degree of change in EPSP slope at the 50% maximal point with p < 0.05 considered significant. An example of IO curve analysis is shown as Supplemental Fig. 1. Data in figures for physiological studies are from continuous monitoring of EPSPs at low frequency during the course of experiments and thus may differ from numerical results described in the text, which represent analyses based on comparison of input–output curves. Statistics were performed using commercial software (GraphPad Prism 9.2.0, GraphPad Software, La Jolla California). For comparisons of LTP results among 0 ppm, 0.4 ppm and 4 ppm Roundup, data were analyzed by one-way analysis of variance (ANOVA) followed by Tukey’s multiple comparison test. For comparisons of LTP results with 100 µM glyphosate, data were analyzed by one-way analysis of variance (ANOVA) followed by Dunnet’s multiple comparison test and compared to 100 µM glyphosate. For non-normally distributed data analysis of one-trial learning after in vivo injection of glyphosate, Kruskal–Wallis test followed by Dunn’s multiple comparison test was used.

### Ethical approval and consent to participate

The animals used in this study were housed in approved facilities at Washington University. Animal use followed National Institute of Health (NIH) guidelines and was approved by the Washington University Institutional Animal Care and Use Committee (IACUC).

## Results

### Glyphosate inhibits hippocampal LTP

**Figure 1:**
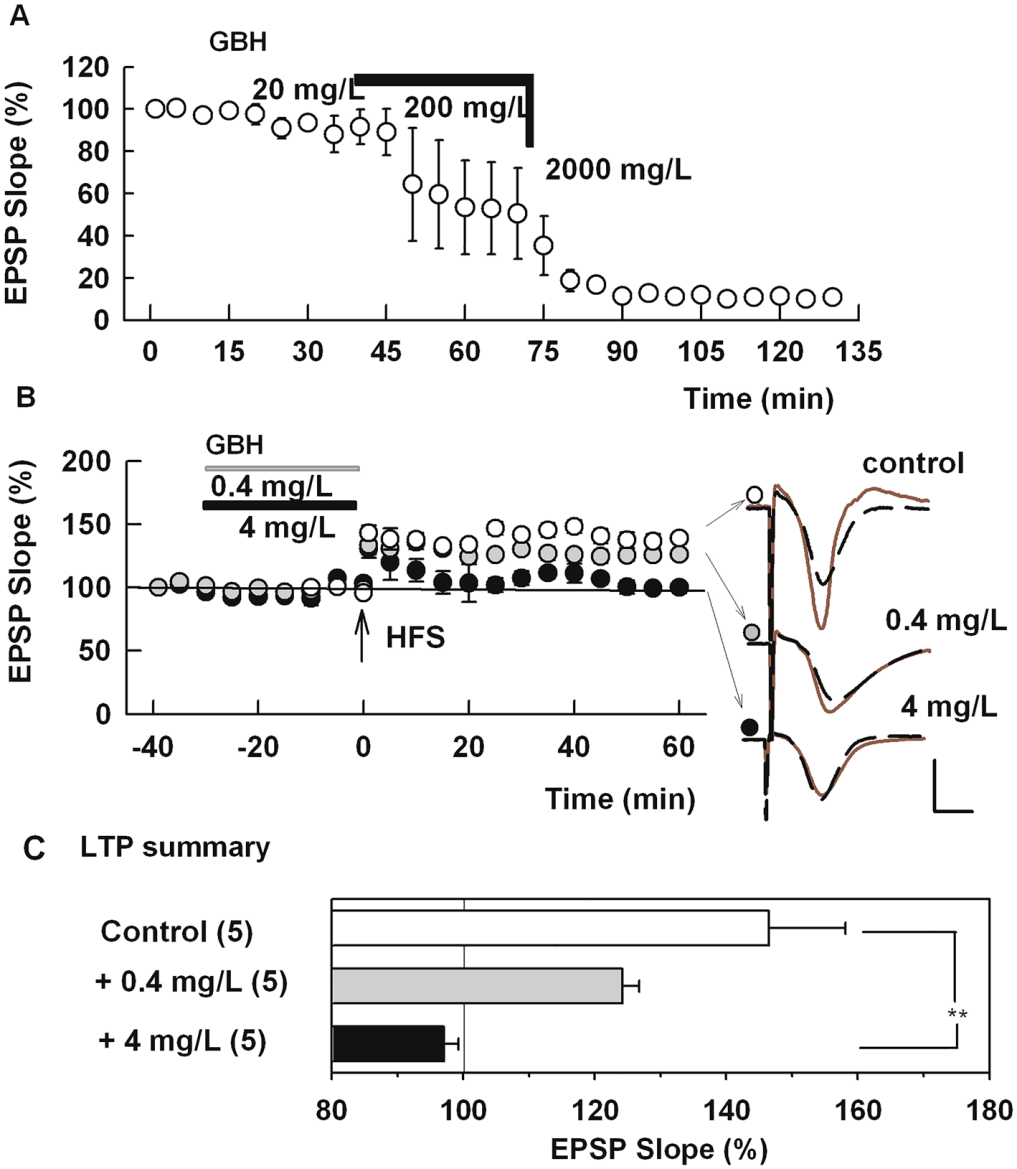
GBH (Roundup) suppresses basal synaptic transmission and LTP in the CA1 region of hippocampal slices. (**A**) In 3 slices, the concentration of GBH was raised stepwise every 30 min. EPSPs, evoked by stimulation of the Schaffer collateral pathway, were not altered by 20 mg/L but were completely suppressed at 2000 mg/L GBH, which is equivalent to 840 mg/L of glyphosate. The suppression did not reverse after 30 min of GBH washout. (**B**) In control slices (open circles) and slices preincubated with 0.4 mg/L GBH (gray circles), but not in slices preincubated with 4 mg/L GBH for 2–4 h (closed circles), LTP was observed after a single 100 Hz × 1 s HFS (arrow). Traces to the right of the graph in this and subsequent figures show representative EPSPs during baseline recordings (black dashed traces) and 60 min following HFS (red traces). Calibration bar: 1 mV, 5 ms. (**C**) LTP results from IO curve calculation. () shows N. **P < 0.001.

In initial experiments, we exposed hippocampal slices to increasing doses of Roundup (a glyphosate-based herbicide, GBH) to determine whether it affects basal synaptic transmission in the CA1 region. When GBH was perfused in increasing concentrations every 30 min, EPSPs were suppressed by high concentrations of GBH and this suppression did not recover within 30 min after wash out of the herbicide (N = 3, Fig. 1A). Although 20 ppm or less of GBH did not affect baseline EPSPs, administration of a 100 Hz × 1 s HFS failed to induce LTP in slices pretreated with 4 ppm GBH for 2–4 h (97.1 ± 2.2%, N = 5, Fig. 1B). This is statistically smaller than matching control LTP in the absence of GBH (146.5 ± 11.6%, N = 5, P = 0.0006). The degree of LTP induced in slices pretreated with 0.4 ppm GBH for 2–4 h (124.2 ± 2.6%, N = 5, Fig. 1B) is not statistically different from control LTP (P = 0.851) but is larger than changes observed at 4 ppm (P = 0.0351). LTP results are summarized in Fig. 1C.

**Figure 2:**
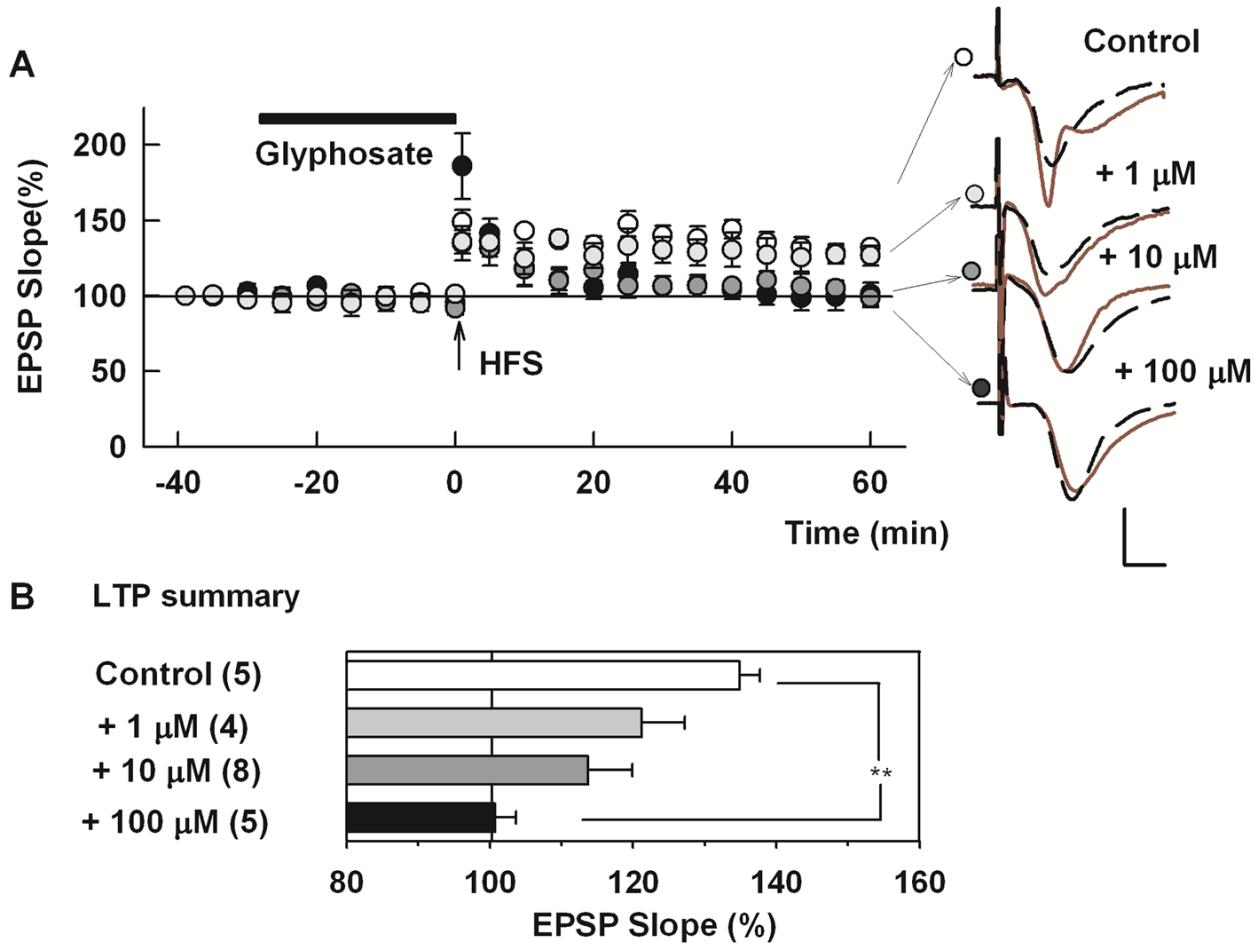
Glyphosate acutely inhibits LTP in the CA1 region. (**A**) In the absence of glyphosate (open circles), HFS (arrow) successfully induced LTP. Acute administration of 1 µM (light gray circles) or 10 μM glyphosate (dark gray circles) produced variable suppression of LTP induction. One hundred μM glyphosate (black circles) inhibited LTP induction completely and reliably. Traces to the right of this graph show representative EPSPs as in Fig. 1. Calibration bar: 1 mV, 5 ms. (**B**) LTP results from IO curve calculation. () shows N. **P < .001.

Because glyphosate is the main ingredient in GBH, we next examined whether glyphosate itself alters basal transmission or LTP induction. In the absence of glyphosate, HFS consistently induced LTP in control slices (Control LTP: 135.0 ± 2.8% of baseline measured 60 min after HFS, N = 5, Fig. 2A).

When administered for 30 min, neither 1 µM, 10 µM nor 100 µM glyphosate had a significant effect on basal synaptic responses. However, 30 min administration of 1 µM or 10 μM glyphosate dampened LTP induction with some variability among slices (121.2 ± 6.0%, N = 4, and 113.8 ± 6.2%, N = 8, respectively, Fig. 2A). At 100 μM, glyphosate completely and reliably suppressed LTP induction (100.7 ± 4.5%, N = 5, P = 0.0002 vs control LTP, Fig. 2A). We also observed that a lower concentration of glyphosate inhibited LTP when slices were pretreated with 1 µM glyphosate for 2–4 h (101.7 ± 4.7%, N = 7), though LTP induction was not altered by similar administration of 0.1 µM glyphosate (128.0 ± 2.7%, N = 5, Supplemental Fig. 2). In subsequent experiments, we focused on acute administration of 100 µM glyphosate. Although acute administration of 100 µM glyphosate is not equivalent to environmental exposures, we used this paradigm with specific inhibitors of inflammation to elucidate mechanisms underlying LTP inhibition by glyphosate in the following studies. LTP results are summarized in Fig. 2A.

### Glyphosate inhibits LTP and learning via pro-inflammatory signaling

**Figure 3:**
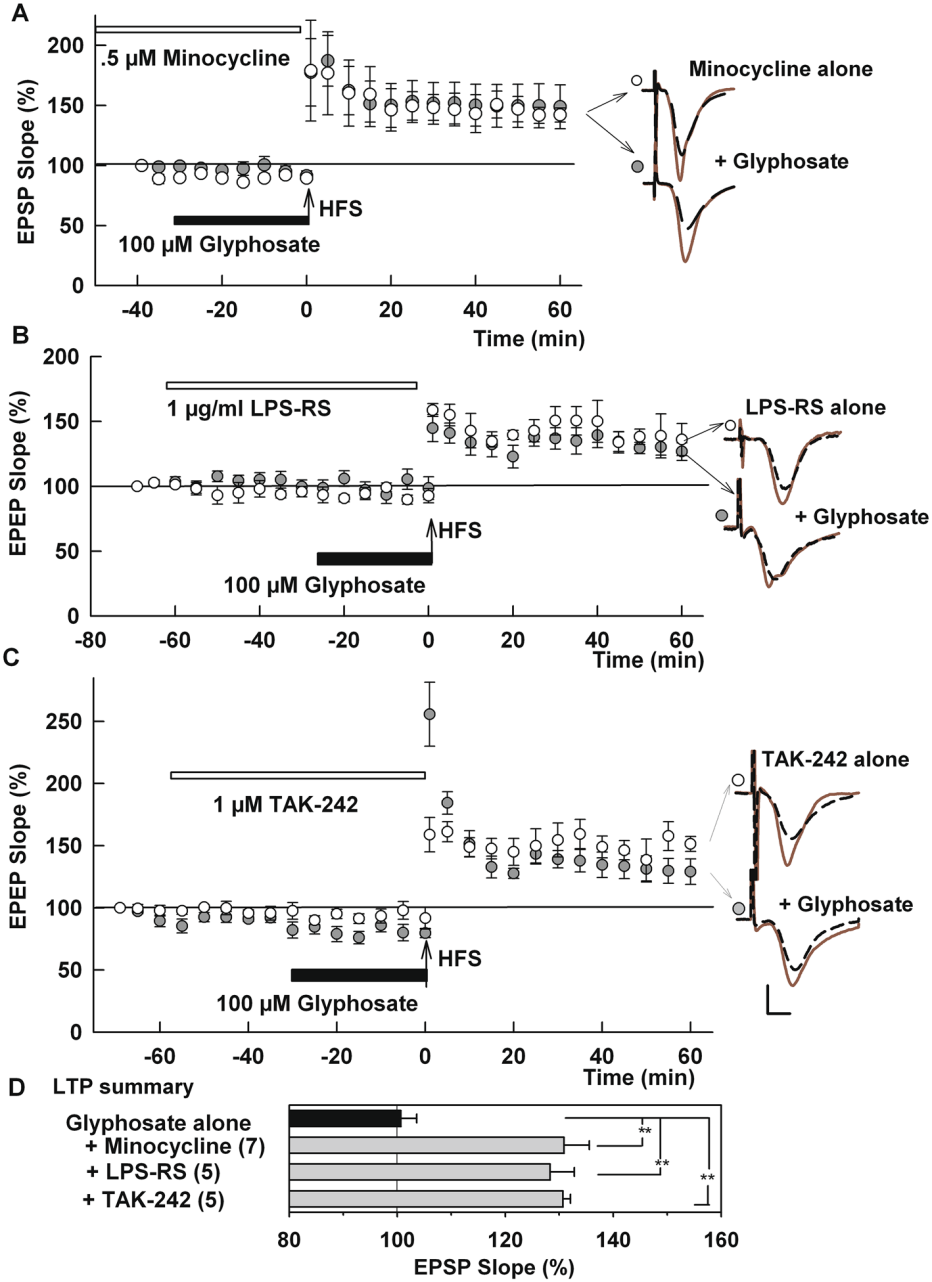
Modulators of microglial neuroinflammation overcome effects of 100 µM glyphosate on LTP. (**A**) Prolonged administration of minocycline (white bar), an inhibitor of microglia, allows LTP induction after HFS (arrow) in spite of the presence of 100 µM glyphosate (black bar) (N = 5). Open circles show minocycline alone (N = 3). (**B**) LPS-RS (white bar), another TLR4 antagonist, overcomes inhibitory effects of 100 µM glyphosate on LTP induction (N = 5). Open circles show LPS-RS alone (N = 3). (**C**) TAK-242 (white bar), a TLR4 antagonist, also allowed LTP induction (N = 5). Open circles show TAK-242 alone (N = 4). Traces show representative EPSPs. Calibration: 1 mV, 5 ms. (**D**) LTP results from IO curve calculation. () shows N. **P < .001, *P < 0.005.

Based on a recent study indicating that glyphosate evokes inflammatory responses^19^, we examined whether microglia are involved in the adverse effects on LTP. Previous studies indicate that glyphosate morphologically modifies microglia to an active form^31^, and perinatal exposure to glyphosate activates or increases microglia in the brains of offspring^32,33^. For our acute experiments, we used minocycline, an agent that is known to inhibit microglia and to have anti-inflammatory effects^34,35^. We found that pre-treatment with minocycline overcame the inhibitory action of glyphosate. In slices pre-incubated with 0.5 µM minocycline, HFS readily induced LTP in the presence of 100 µM glyphosate (131.0 + 4.7%, N = 7, P = 0.0004 vs 100 µM glyphosate alone, Fig. 3A), supporting a role for microglia in the acute effects of glyphosate. Minocycline alone had no effect on LTP (131.4 ± 1.9%, N = 3; Fig. 3A).

Because the toll-like receptor 4 (TLR4) signaling complex plays a key role in activation of microglia by pro-inflammatory stimuli^36^, we next examined a role for TLR4 in the effects of glyphosate using inhibitors of this receptor. LPS-RS, a TLR4 inhibitor that antagonizes the receptor via two distinct mechanisms^37,38^, overcame the inhibitory effects of glyphosate on LTP at a concentration of 1 μg/ml (128.3 ± 1.4%, N = 5, P = 0.0034 vs. glyphosate alone, Fig. 3B). We also found that a more specific TLR4 antagonist, TAK-242 (1 µM), completely prevented the effects of 100 µM glyphosate on LTP induction (130.7 ± 4.5%, N = 5, P = 0.0013 vs. 100 µM glyphosate alone, Fig. 3C). Post-tetanic potentiation was robust with large variations in the presence of TAK-242 with glyphosate but was not so with TAK-242 alone. Neither LPS-RS (138.3 ± 1.5%, N = 3) nor TAK-242 (138.7 ± 3.2%, N = 4) alone had an effect on LTP (Fig. 3B,C). LTP results are summarized in Fig. 3D.

**Figure 4:**
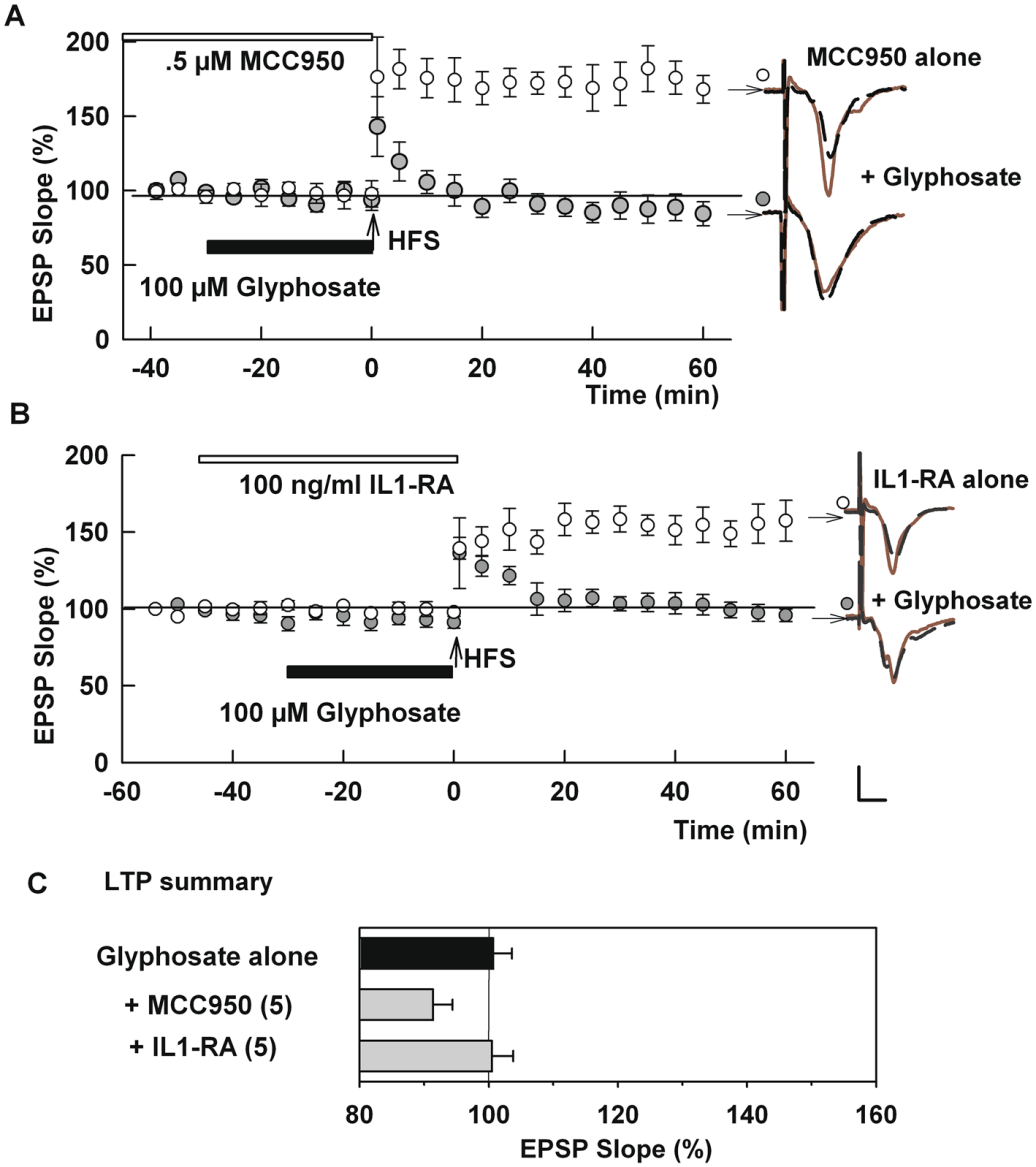
An NLRP3 inhibitor and IL-1 receptor antagonist failed to overcome effects of glyphosate on LTP. (**A**) Administration of 0.5 μM MCC950, an inhibitor of NLRP3 for 2–4 h (white bar) prior to HFS (arrow) did not prevent LTP inhibition in the presence of 100 µM glyphosate (black bar) (N = 5). Open circles show MCC950 alone (N = 3). (**B**) Similarly, in the presence of 100 ng/ml interleukin-1 receptor antagonist (IL-1Ra) (white bar), glyphosate still blocked LTP induction (N = 5). Open circles show IL-1Ra alone (N = 3). Traces show representative EPSPs. Calibration: 1 mV, 5 ms. (**C**) LTP results from IO curve calculation. () shows N.

One of the major consequences of TLR4 activation is stimulation of the NLRP3 inflammasome and release of the pro-inflammatory cytokine, interleukin-1 (IL-1)^39,40^. However, we observed that 100 μM glyphosate still inhibits LTP induction in slices pre-incubated with 0.5 μM MCC950, an inhibitor of NLRP3 (93.4.8 ± 3.0%, N = 5, P = 0.8957 vs. glyphosate alone, Fig. 4A). MCC950 alone, in the absence of glyphosate, did not alter LTP induction (152.7 ± 4.0%, N = 3). Similarly, 100 μM glyphosate still inhibits LTP in the presence of 100 ng/ml interleukin-1 receptor antagonist (IL-1Ra) (106.5 ± 3.3%, N = 5, P > 0.999 vs. glyphosate alone, Fig. 4B). IL-1Ra alone, in the absence of glyphosate, did not alter LTP induction (139.0 ± 7.0%, N = 3). These negative results suggest that glyphosate dampens synaptic plasticity independently from NLRP3 activation. LTP results are summarized in Fig. 4C.

**Figure 5:**
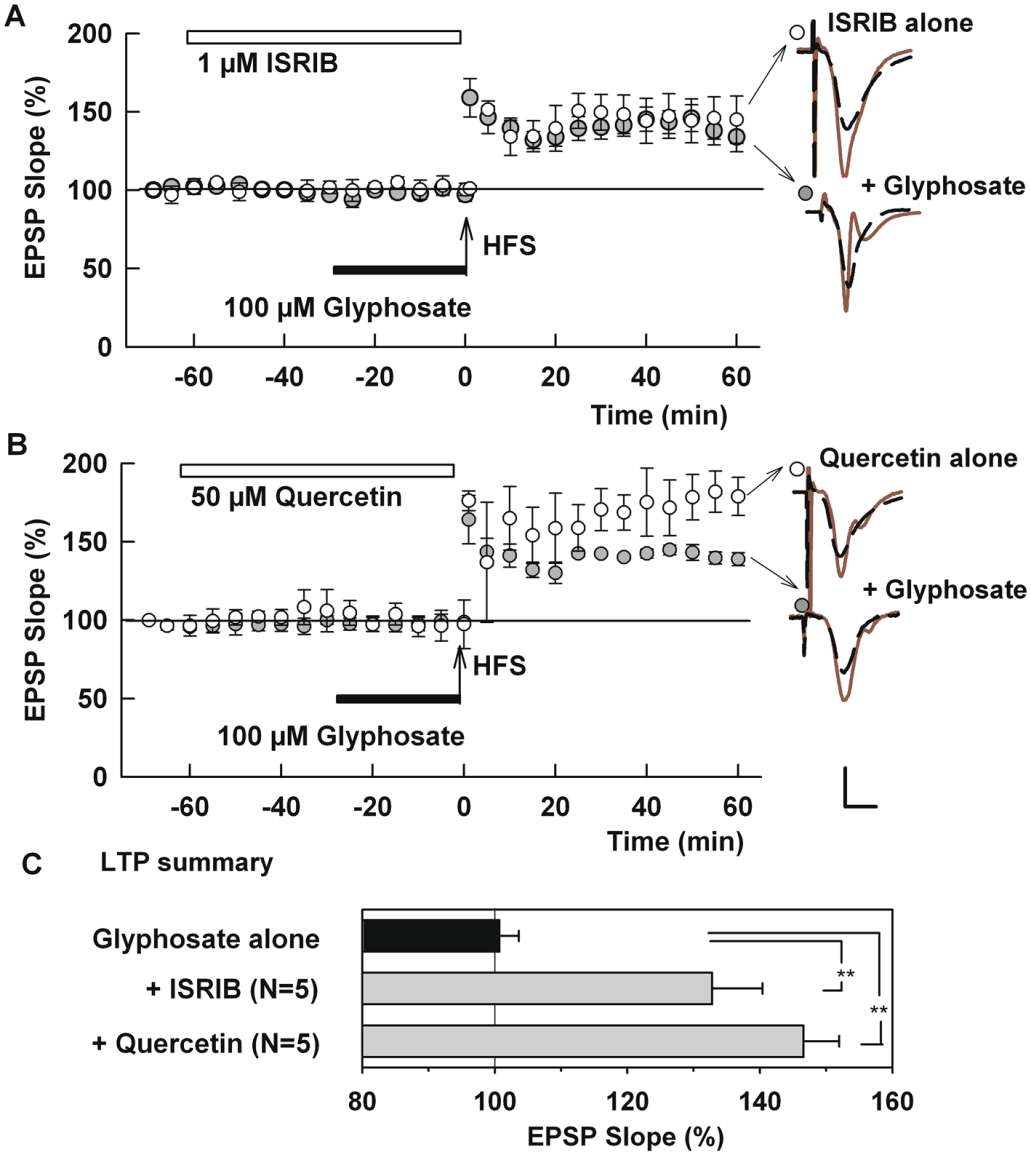
Inhibitors of cellular stress responses overcome the effects of glyphosate on LTP. (**A**) Administration of 1 μM ISRIB (white bar) prior to HFS (arrow) allowed LTP induction in spite of the presence of 100 µM glyphosate (black bar) (N = 5). Open circles show ISRIB alone (N = 3). (**B**) Similarly, in the presence of 50 µM quercetin (white bar), glyphosate failed to block LTP induction (N = 5). Open circles show quercetin alone (N = 3). Traces show representative EPSPs. Calibration: 1 mV, 5 ms. (**C**) LTP results from IO curve calculation. () shows N. ** P < .001.

TLR4 signaling and microglial activation are also known to stimulate intracellular stress responses, which, in turn, can adversely modulate induction of synaptic plasticity^21,22^. To test this possibility, we used ISRIB, a specific inhibitor of the integrated stress response that reverses the effects of eIF2 phosphorylation and preserves memory functions^41^. In the presence of 1 µM ISRIB, glyphosate failed to inhibit LTP induction (132.0 ± 7.7%, N = 5, P = 0.0008 vs. glyphosate alone, Fig. 5A). We also examined the effects of quercetin, a flavonoid that attenuates inflammatory processes through inhibition of endoplasmic reticulum stress^42^. At 50 µM, quercetin also allowed robust LTP induction in the presence of 100 µM glyphosate (146.6 ± 5.4%, N = 5, P < 0.0001, Fig. 5B). Neither ISRIB (148.4 ± 17.1%, N = 3) nor quercetin (163.7 ± 7.1%, N = 3) significantly altered LTP when administered alone (Fig. 5A,B). LTP results are summarized in Fig. 5C.

**Figure 6:**
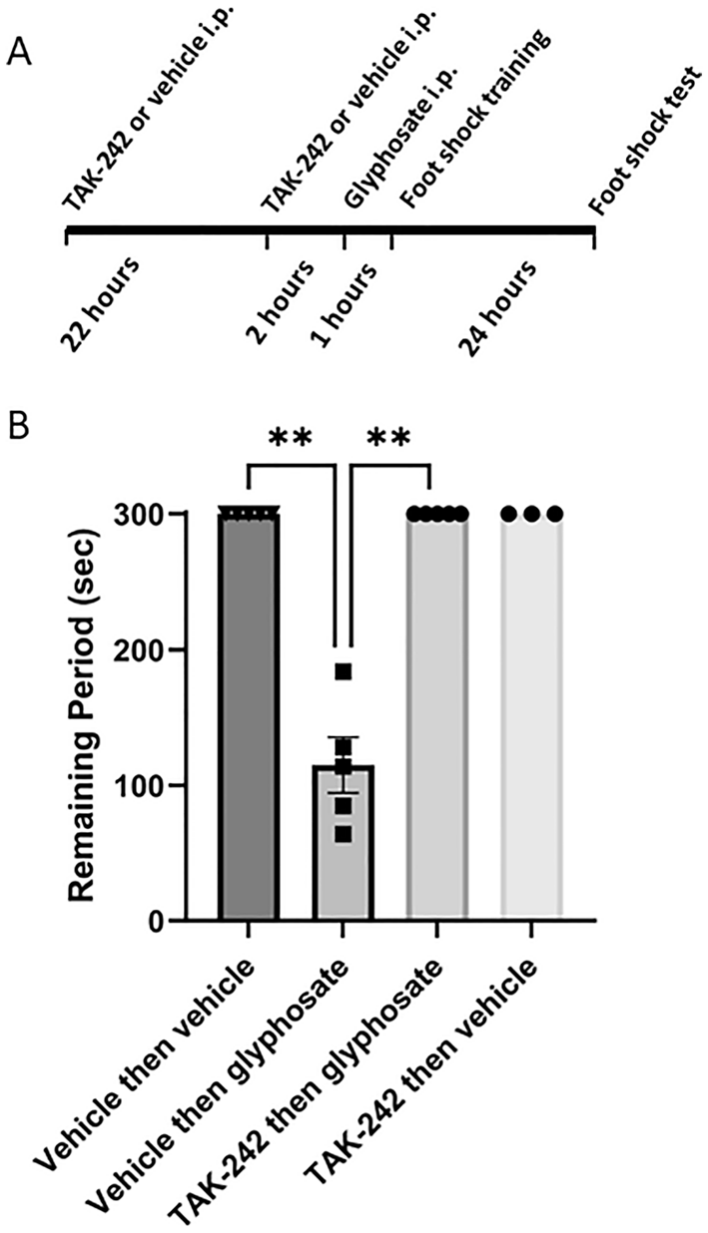
Effects of glyphosate in vivo. (**A**) Experimental design of the exposures to TAK-242 and glyphosate in male rats. (**B**) Intraperitoneal injection of glyphosate (16.9 mg/kg ip) one day prior to inhibitory avoidance training results in a defect in learning as manifest by rats more readily leaving the lit chamber to enter the dark chamber where they had received a foot shock one day previously. Injection of TAK-242 (3 mg/kg, 26 h and 2 h before glyphosate administration) prevented the learning deficit. TAK-242 alone had no effect on learning. **p < 0.01 by Dunn’s multiple comparison test.

To determine whether effects observed in ex vivo hippocampal slices translate into changes in learning and memory, we also examined the effects of glyphosate on a one-trial inhibitory avoidance task that has been linked previously to hippocampal LTP^20^. Glyphosate was injected at a dose of 16.9 mg/kg, i.p. 24 h before conditioning (see Fig. 6A for experimental paradigm). However, glyphosate treatment had marked acute effects on performance in one-trial learning compared to saline-treated controls when tested 24 h after conditioning. The glyphosate-induced defect in learning was manifest by rats more readily entering the dark chamber where they had been shocked during training, whereas saline-treated controls remained in the lit compartment for the full duration of the 300 s trial (P = 0.0045 by Dunn’s test, N = 5, Fig. 6B).

The adverse effects of glyphosate on learning were completely prevented by pretreatment with the TLR4 antagonist, TAK-242. In rats treated with TAK-242 (3 mg/kg i.p. twice), glyphosate had no effect on one-trial learning (P = 0.0045 vs. glyphosate alone by Dunn’s test, N = 5, Fig. 6). Visible changes in gait and coordination were not observed, but depression in weight gain was observed in rats treated with glyphosate alone (Supplemental Fig. 3).

## Discussion

The primary mechanism of glyphosate in plants involves disruption of the shikimate pathway. Because this pathway is absent in animal cells, it has long been held that glyphosate is not harmful to animals. However, in 2015, the World Health Organization’s International Agency for Research on Cancer identified glyphosate as a probable human carcinogen^43^. Although the carcinogenicity of glyphosate is still debated^44^, there are other concerns with this environmental agent. In particular, there are now concerns that the CNS is one of the targets of glyphosate^45^. Parkinson’s disease (PD) was observed in a woman after chronic occupational exposure to GBH^11^, and in rats even shorter exposure to glyphosate alters dopaminergic systems^9,16^. Additionally, a possible link with autism is speculated based on epidemiological data^46^, and maternal exposure to glyphosate results in autism-spectrum disorder (ASD)-like behaviors in murine offspring^8,43^. Excitotoxicity in the CNS is also possible because CSF levels of aspartate and glutamate double within a day after a single oral dose of GBH in rats^47^. In mice, intranasal exposure to GBH results in anxiogenic behaviors^48^. Moreover, oral administration of 250–500 mg/kg GBH results in memory impairment in mice observed as decreased latency in a passive avoidance test^49^.

In the present study, we first tested if GBH itself alters basal synaptic function and found that EPSPs were acutely depressed by 200 mg/L GBH, a level that is equivalent to 82 mg/L or about 500 µM glyphosate. We also found that LTP, a mechanism of learning and memory, was completely disrupted by preincubation of slices with 4 mg/L GBH, which corresponds 1.6 mg/L glyphosate or roughly 10 µM glyphosate. These finding are well paralleled by our studies using glyphosate itself, which inhibited LTP at micromolar levels (Fig. 2 and Supplemental Fig. 2). This level is higher than concentrations detected in healthy human urine samples. For example the maximal concentration in urine from young individuals in Germany is 11.1 µg/L^50^. However, it should be noted that only 1% of glyphosate is secreted in urine^4^, and levels in the circulation could be higher than levels in urine.

In our studies, we hypothesized that glyphosate in GBH causes neuroinflammation to impair cognitive function. It has been recently shown that oral administration of glyphosate to mice (125, 250 and 500 mg/kg for 14 days) elevates glyphosate levels (10–50 ng/mg) and tumor necrosis factor-ɑ (TNFα) in the brain^19^. This study has two important implications: orally administered glyphosate infiltrates the CNS and elevates pro-inflammatory cytokines in the CSF. The aforementioned case of GBH ingestion^18^ also suggests that GBH may trigger inflammation in the CNS. Microglia are major contributors to neuroinflammation. Consistent with this, LTP was successfully induced in the presence of glyphosate when hippocampal slices were pretreated with minocycline, an inhibitor of microglia. The inhibitory effect of glyphosate on LTP induction at least partially shares mechanisms with LPS and acrylamide, both of which induce neuroinflammation^21,22^. The ability of LPS-RS to overcome effects of glyphosate suggests that glyphosate behaves like LPS in the CNS. Moreover, we observed that TAK-242, a selective TLR4 antagonist, clearly overcomes the effects of glyphosate in both LTP and behavioral experiments, suggesting that activation of TLR4 is pivotal for glyphosate to disrupt the CNS. We selected TAK-242 for our behavioral studies because TAK-242 has been widely used to control neuroinflammation in rodents^29,30,51^. In addition, TAK-242 is useful to treat systemic inflammation in animal models of sepsis^52,53^. In our behavioral experiments, the depression in weight gain by glyphosate was attenuated by TAK-242, suggesting that glyphosate also induces systemic inflammation mediated by TLR4 (Supplemental Fig. 3).

Although LTP induction was impaired by acute administration of 10 µM glyphosate, we used 100 µM glyphosate for our experiments because it consistently and completely blocked LTP induction, allowing us to determine mechanisms underlying its neurotoxicity. With this experimental paradigm, we observed that TAK-242 efficiently allows LTP induction in the presence of glyphosate. However, 100 µM glyphosate could be excessive and obscure other contributing mechanisms. Consistent with this, we were surprised that MCC950, a reliable NLRP3 inhibitor, failed to overcome the inhibitory effect of glyphosate on LTP induction because MCC950 effectively overcomes the LTP inhibiting effects of acrylamide, another environmental toxin^21,22^. The failure of MCC950 does not necessarily preclude a role for NLRP3 but the discrepancy may imply that glyphosate activates pro-inflammatory pathways in a manner different from other toxins.

In contrast to MCC-950, we found that the effects of glyphosate can be attenuated by other cellular stress inhibitors. Because the ISR contributes to the pathogenesis of memory impairment and neurodegeneration accompanied by inflammation, systemic inhibition of ISR by ISRIB can reverse memory deficits^54^. In the current study, ISRIB and quercetin successfully prevented the inhibitory effect of glyphosate on LTP induction.

Although it is difficult to prevent GBH exposure as evidenced by the observation that glyphosate is detected in the urine of nearly all (99.8%) of the French population in one study^2^, it is important to identify measures to prevent its neurotoxicity. These measures may include organic diets^55^ and also diets that dampen cellular stress responses. Quercetin, a flavonoid, attenuates inflammation by inhibition of endoplasmic reticulum stress^42^. Interestingly, hepatotoxicity induced by sub-chronic administration of glyphosate in rats is reportedly attenuated by simultaneous administration of quercetin^56^. Moreover, it has been reported that quercetin overcame the decrease of reduced glutathione levels and increase in reactive oxygen species in the mouse hippocampus after sub-chronic exposure to a GBH^57^. Consistent with these reports, quercetin was effective in allowing LTP in the presence of glyphosate in our study. Regular dietary intake of quercetin in vegetables such as onions could help prevent neuroinflammation triggered by GBH if these vegetables are not contaminated with the herbicide.

In this study, we focused on direct neurotoxic aspects of glyphosate and found that glyphosate activates microglia via TLR4 and triggers cellular stress to impair hippocampal plasticity and learning. However, the neurotoxicity of GBH may not be limited to the direct actions of glyphosate. The GBH, Roundup, uses polyethoxylated tallow amine (POEA) as a surfactant and POEA can also contribute to toxicity^58^ because POEA is a strong inducer of ER stress^59^. Gut microbiota dysbiosis by glyphosate also may result in neuronal impairment^6^ because block of the shikimate pathway impacts microbiota. Furthermore, aminomethylphosphonic acid (AMPA), one of glyphosate’s main metabolites, may have additional actions. Thus, the neurotoxicity of GBH is likely more complicated and perhaps more severe than the results observed in the present study.

### Supplementary Information

Supplementary Legends.

Supplementary Figure 1.

Supplementary Figure 2.

Supplementary Figure 3.

## Data Availability

The datasets used and/or analyzed during the current study available from the corresponding author on reasonable request.

## Abbreviations

ACSF: Artificial cerebrospinal fluid
AMPA: Aminomethylphosphonic acid
ANOVA: Analysis of variance
ASD: Autism spectrum disorder
BBB: Blood brain barrier
CNS: Central nervous system
EPSP: Excitatory postsynaptic potentials
GBH: A glyphosate-based herbicide
HFS: High frequency stimulation
IACUC: Institutional Animal Care and Use Committee
IL-1: Interleukin-1
IO: Input–output
i.p.: Intraperitoneally
LPS: Lipopolysaccharide
LPS-RS: Lipopolysaccharide from *Rhodobacter sphaeroides*
IR-Ira: Interleukin-1 receptor antagonist
ISR: Integrated stress response
ISRIB: Integrated stress response inhibitor
LTP: Long-term potentiation
mtROS: Mitochondrial reactive oxygen species
NIH: National Institute of Health
NLRP3: NOD-, LRR- and pyrin domain-containing protein 3
P: Postnatal day
PD: Parkinson’s disease
TNF-α: Tumor necrosis factor-ɑ
