# Supplementary material for: The herbicide glyphosate inhibits hippocampal long-term potentiation and learning through activation of pro-inflammatory signaling
Source: Sci Rep. 2023 Oct 21;13:18005. doi: 10.1038/s41598-023-44121-7 (PMC10590375; doi:10.1038/s41598-023-44121-7)
Supplement: Supplementary file 1 — Supplementary Legends. [file 41598_2023_44121_MOESM1_ESM.pdf]

Supplemental Figure Legends

Supplemental Figure 1.Typical input-output (IO) curves for EPSP slopes generated from 6 different stimuli. Black circles represent responses obtained before HFS whereas open circles represent responses 60 min after HFS. Arrows show procedures to calculate changes following HFS: From 100% maximal points, 50% amplitude (a) is determined and then corresponding amplitude obtained 60 min after HFS (a+b) is found. “b” indicates the increase and “b/(a+b) x 100% ” represents LTP magnitude.

Supplemental Figure 2. Preincubation of slices with glyphosate at 1 μM but not 0.1 μM interrupts LTP induction. A. HFS induces LTP in slices pretreated with 0.1 μM glyphosate (gray circles) but failed to induce LTP in slices pretreated with 1 μM glyphosate (black circles). B. LTP summary.

Supplemental Figure 3. Body weight changes one day after injection of glyphosate. This figure corresponds to Figure 6.
