# Supplementary figures and images for: The herbicide glyphosate inhibits hippocampal long-term potentiation and learning through activation of pro-inflammatory signaling
Source: Sci Rep. 2023 Oct 21;13:18005. doi: 10.1038/s41598-023-44121-7 (PMC10590375; doi:10.1038/s41598-023-44121-7)

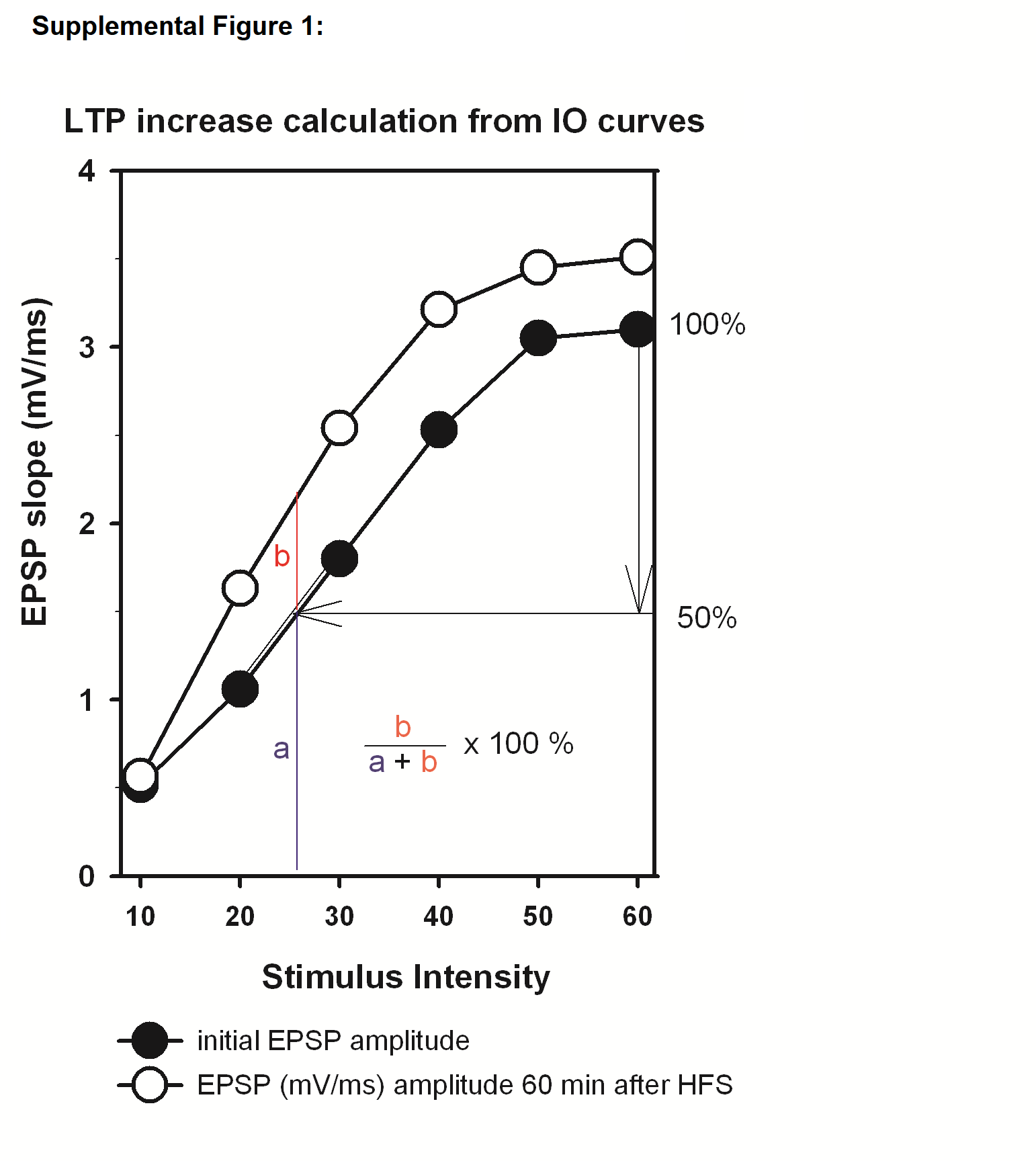

Supplement: Supplementary file 2 — Supplementary Figure 1. [file 41598_2023_44121_MOESM2_ESM.tif]

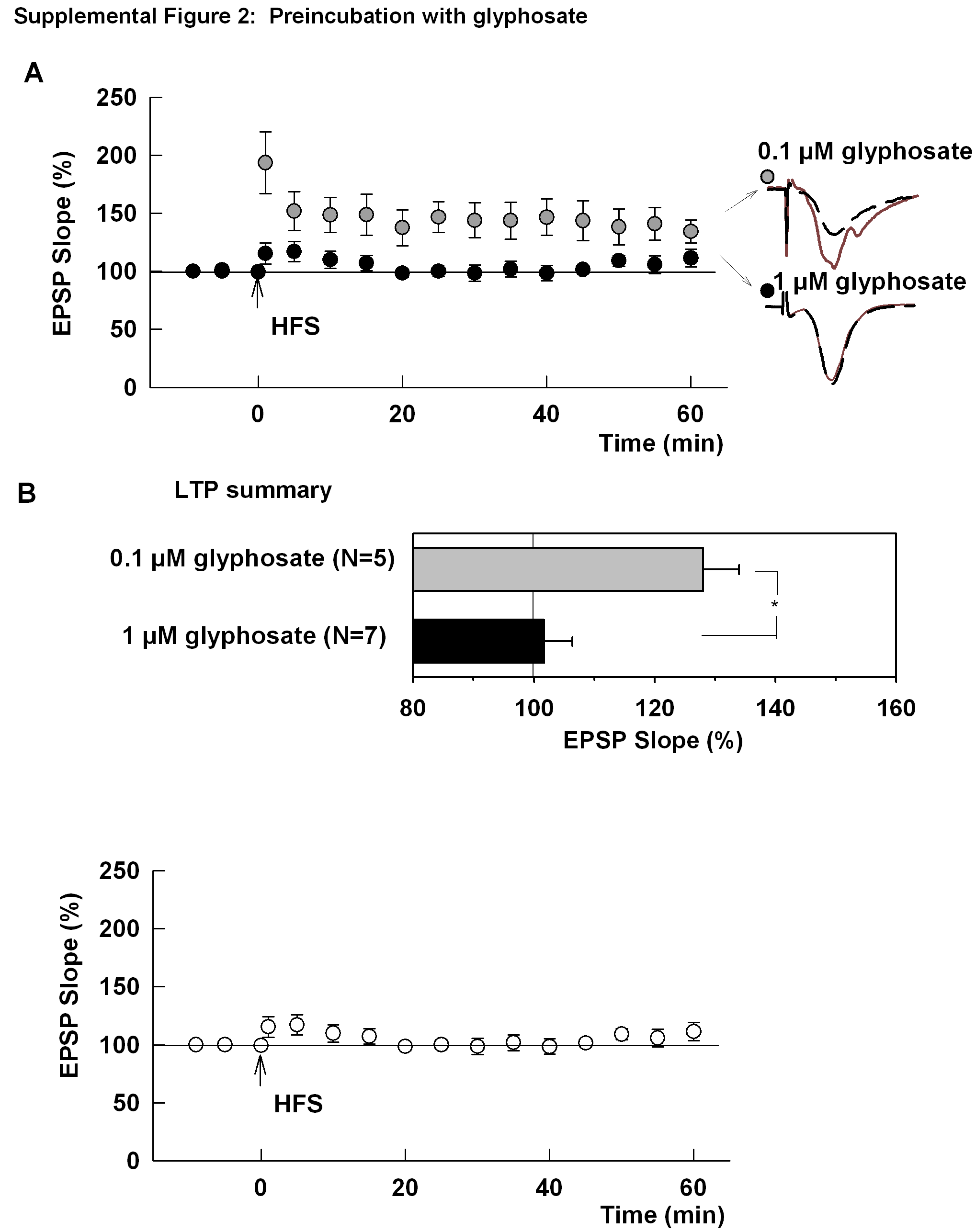

Supplement: Supplementary file 3 — Supplementary Figure 2. [file 41598_2023_44121_MOESM3_ESM.tif]

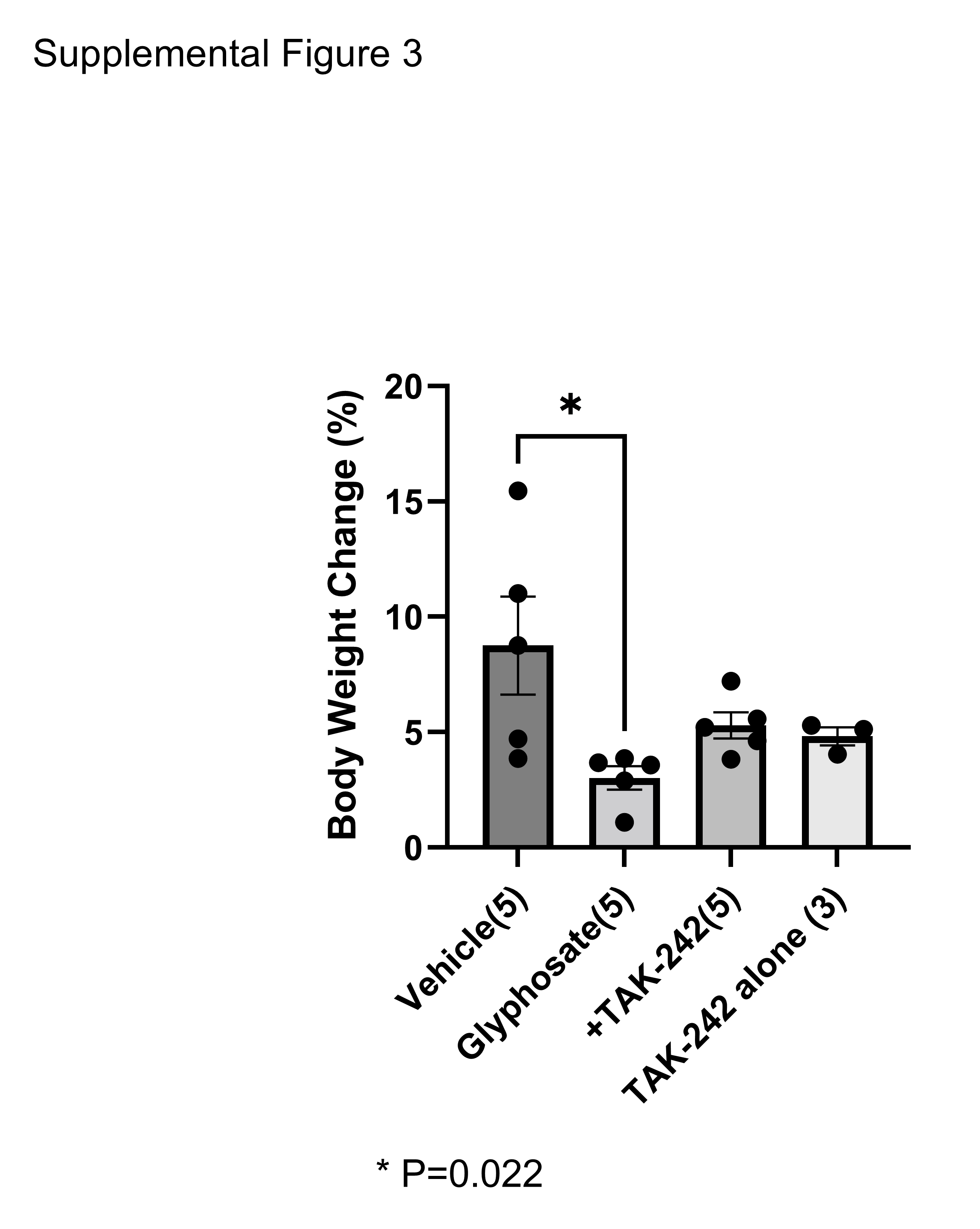

Supplement: Supplementary file 4 — Supplementary Figure 3. [file 41598_2023_44121_MOESM4_ESM.tif]
